# Motivators and Facilitators of Fruit and Vegetable Intake in Tongan Adults

**DOI:** 10.3390/nu17091510

**Published:** 2025-04-29

**Authors:** Emily Mitchell, Crystal Áke, Steven Underhill, Sarah Burkhart

**Affiliations:** 1 School of Health, University of the Sunshine Coast, Locked Bag 4, Maroochydore, QLD 4558, Australia; sburkhar@usc.edu.au; 2 Australian Centre for Pacific Islands Research, University of the Sunshine Coast, Locked Bag 4, Maroochydore, QLD 4558, Australia; sunderhi@usc.edu.au; 3 Mainstreaming of Rural Development Innovation Tonga Trust, Hala Vaha’akolo, Havelu, Nuku’alofa P.O. Box 966, Tonga; c.ake@morditonga.to

**Keywords:** fruits and vegetables, motivators, facilitators, Tonga, nutrition, health, Pacific Islands

## Abstract

Background/Objectives: Changes to food systems and food environments across the Pacific Islands region have influenced dietary behaviors. While dietary intake is limited, it is believed that fruit and vegetable consumption is low within these populations. Given the benefits of fruit and vegetable consumption, this exploratory study aimed to identify the motivators and facilitators of fruit and vegetable intake in a subset of Tongan adults. Methods: Semi-structured, 30 min interviews were conducted in Tongan with 12 participants (5 fieldworkers and 3 female community members who were interviewed separately and 4 women who took part in a small group interview). The responses were translated into English and were analyzed using thematic analysis. Results: Most participants thought that low fruit and vegetable consumption was of concern in Tongan communities. All participants agreed that diverse fruit and vegetable consumption is a priority for Tongan people and that communities are motivated to consume more fruits and vegetables. A total of 12 themes were derived from the results: 6 motivational and 6 facilitating, including determinants such as dietary patterns (taste and culture), community engagement/working in groups, and financial factors. Conclusions: While using a small sample size from Tongatapu, this provides a valuable insight from the Tongan communities’ perspectives on motivators and facilitators of fruit and vegetable consumption. The results offer policymakers, government, and NGOs contextually relevant and up-to-date data that can be used when developing health and nutrition programs.

## 1. Introduction

In recent decades [1,2], rapid socioeconomic changes, globalization, and the impacts of climate change have significantly altered the lifestyles, food choices, and, consequently, health behaviors of South Pacific Islander populations. A widespread and escalating triple burden of malnutrition (the co-existence of overnutrition, undernutrition, and micronutrient deficiencies) [3] and diet-related non-communicable diseases (DR-NCDs) are now prevalent across this region [3,4]. These challenges have provided impetus to explore opportunities to support more nutritious food choices and dietary behaviors.

Before 1970 [2], Pacific peoples were largely dependent on local crops sourced from subsistence and semi-subsistent production systems, as a source of food and nutrition [2,4,5]. Whilst subsistence farming is still practiced across the Pacific Islands region, an accelerated nutrition transition (a shift in human activity and dietary patterns resulting in nutritional outcomes) [6] has created a reliance on and preference for highly processed, imported foods, which are typically high in sugar, salt, and saturated fat [7,8]. The introduction of these foods to the South Pacific has shaped current food systems, supply chains, food environments, and consumer behaviors, leading to poor diet diversity and the creation of obesogenic environments [2,9]. The saturation of these highly processed, inexpensive, and imported foods within the food environment makes healthy food choices even more challenging.

The Kingdom of Tonga (Tonga) is an archipelago in the Polynesian region of the South Pacific. Tonga’s food system is dependent on small-scale fisheries, small retail outlets, local roadside and in-town markets, and subsistence farming [1] but has also been proliferated by an influx of highly processed, imported foods. Currently, Tonga has some of the highest rates of DR-NCDs in the world [10,11]. Recent data report that 61.3 percent of Tongan males (18+ years) and 79.1 percent of Tongan females (18+ years) are classified as obese [11]. Furthermore, 12.6 percent of Tongan children under the age of five [12,13], and 60 percent of adolescents (13–15 years of age) are reported to be overweight [14,15].

There are limited data on actual dietary consumption in Tonga and more broadly across the Pacific Islands region. However, based on the increased availability and accessibility of highly processed imported foods, and high rates of DR-NCDs, it is plausible to assume that these changes in the food system have decreased diet quality. It is acknowledged that fruit and vegetable consumption is low across the Pacific Islands region [16] due to climatic [17], economic [17], trade [14], and consumer preference [18] reasons. This is also evident in Tonga. For a healthy diet, it is recommended that five servings of fruits and vegetables are consumed daily [19]. Tongan adults have been reported to consume 0.8 servings of fruits and vegetables a day, and only 38.7% of Tongan adolescents are meeting the recommendation [11]. Diets low in fruits and vegetables are more likely to increase the risk of DR-NCDs [20,21]. Fruits and vegetables are nutrient rich, containing folate, vitamins A, C, and E, and minerals such as magnesium and potassium [22] and are a good source of fiber [23]. These compounds can reduce DR-NCDs as they provide the body with health protective antioxidants, and anti-inflammatory properties, and are low in energy, decreasing the probability of weight gain, type II diabetes, hypertension, and obesity [24].

Food choice is a complex decision-making process which is made more difficult in a rapidly changing food environment [25], like that observed in Tonga. Tongans have a strong cultural connection to food, with food playing a significant role in day-to-day life and special occasions [26,27]. It has been reported that Tongan people perceive highly processed foods as a delicacy with many holding cultural significance [28] alongside the belief that larger body sizes indicate a higher social ranking [26,29]. Processed foods (in particular meats) that are higher in price, have been reported to indicate a more affluent economic status [1,18], along with larger portion sizes reflecting love, politeness, and respect [12,30]. Food choice is also heavily influenced by what is easily and readily available [25]. While system-level change is needed to enhance the supply, availability, and affordability of healthy foods, understanding the motivators and facilitators of an individual’s or communities’ choices is crucial for encouraging more nutritious food choice and behavior change.

As the literature is limited on the motivators and facilitators of fruit and vegetable consumption from both the Pacific Islands and Tongan perspectives, further exploration and documentation of this topic is required. Therefore, this exploratory study aimed to identify current behaviors, motivators, and facilitators of fruit and vegetable consumption in Tongan adults.

## 2. Materials and Methods

### 2.1. Location

**Figure 1:**
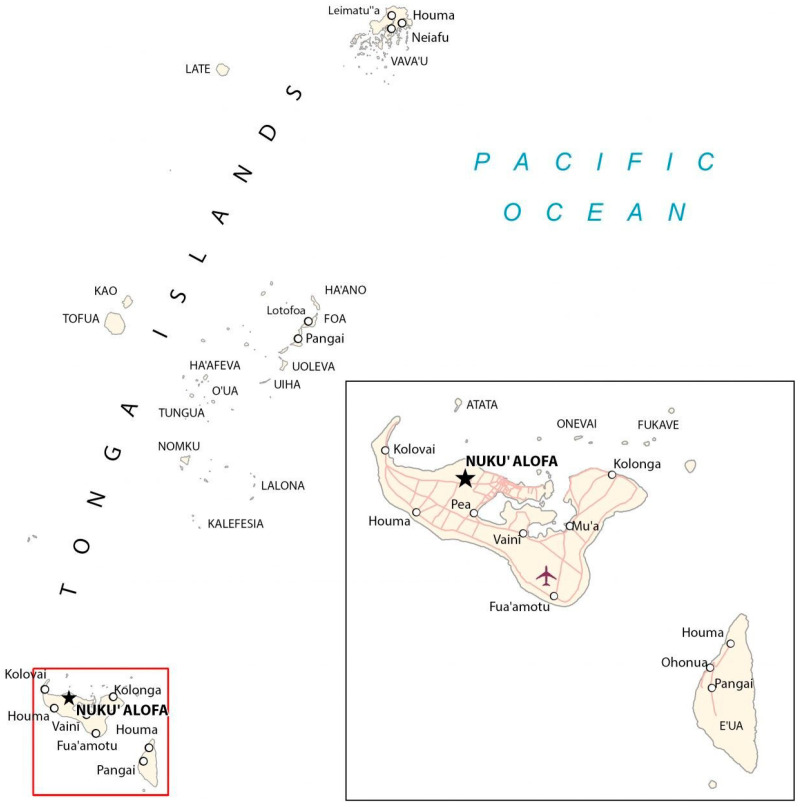
Map of Tonga. This map shows the dispersion of the Tongan islands in the South Pacific region. Featured at a larger scale in the bottom right corner is Tongatapu (**to the left**) and ‘Eua (**to the right**) where the participants were located [34].

Tonga is a middle-income country [31] in the South Pacific Ocean (Figure 1), made up of 169 islands, [32] of which 39 are inhabited [32]. Tonga’s population is 100 179 [33] with 70 percent living on the main island of Tongatapu and 23 percent living in urban areas. The population of Tongatapu is categorized into seven districts (personal communication, 9 August 2023), and within the districts there are multiple communities [7,31].

### 2.2. Participants

This cross-sectional study was conducted between August and September 2023 using semi-structured interviews to explore the motivators and facilitators of fruit and vegetable consumption of Tongan adults. Given the exploratory nature of this study, Tonga’s small population size, the potential participant burden of semi-structured interviews, and the high possibility of data saturation, an initial sample of 12 participants was decided on. Five male fieldworkers from a local rural development NGO (who have experience in development projects that support health and nutrition) and three female members of the community were interviewed individually, and an additional small group interview was conducted with four female community members, with each interview varying from 30 to 60 min in length. The participants were from various communities in Tongatapu, and one participant was from the neighboring island, ’Eua.

Eligible participants were Tongan residents over the age of 18 years. All participants were recruited through convenience sampling facilitated by in-country collaborators via a verbal request and no incentive was provided to participate in this study. Participants were provided with a document outlining the details of this study and what was required from their participation. Verbal consent was given because the interviews were completed in an informal environment, conducted by in-country collaborators; thus, it was easier to collect this information verbally. The survey did not continue if consent was not provided.

Field workers were prioritized for inclusion as they have insights as both a member of a local Tongan community but also have experience in working with community groups to enhance agricultural practices and to improve food and nutrition security. Being in this position of work ensured that these participants had a sound understanding of nutritional terms and fruit and vegetable intake guidelines. Additionally, their daily interaction with community members (with a focus on improving health and access to fruits and vegetables) was used with the intent to capture motivators and facilitators that may not be identified by those working in different sectors, especially those that focus on the treatment of NCDs (e.g., those in health care). Other community members, specifically women, were approached to participate to ensure that both sexes were represented in the data collection. This was conducted as a small group interview based on the participants’ preferences and availability. Measures were in place to limit group thinking during the small group interview; however, it is believed that group thinking was not a factor. The participants were selected by in-country collaborators based on their experience and engagement in past work. Additionally, the local Tongan author stated that the informal environment created a supportive space that encouraged free speech and discussion amongst the participants. The interviewer also identified participants who contributed less during the small group interview and followed up with them directly after the session to ensure their perspectives had been captured. The interviewer used different approaches to ensure the participants understood and had enough time to answer the questions and to provide authentic responses. This study had ethical approval from the researcher’s institution (approval number: A221743).

### 2.3. Data Collection Tool

As this study sought to explore the current behaviors, motivators, and facilitators of fruit and vegetable consumption in Tongan adults, these goals guided the development of the semi-structured interview questions. Current behaviors were considered to include practices that contributed to a low intake of fruits and vegetables and practices that could support increasing intake. Motivators were considered as influences that determine food consumption and food practices (e.g., attitudes, preferences, social norms, etc.), and facilitators were considered as skills and knowledge that influence food behavior (e.g., social support, nutrition knowledge, cooking skills, etc.) [35].

A total of 18 questions (Table 1) were developed by the research team. The questions were tailored to be carried out with either the fieldworkers or women. In-country collaborators conducted the interviews in Tongan, encouraging detailed responses that would likely not have been provided if the interviews had been conducted in English.

### 2.4. Data Analysis

Reflexive thematic analysis was selected based on the data obtained from the interviews and the exploratory nature of this work. As described by Braun and Clarke [36], “Through its theoretical freedom, thematic analysis provides a flexible and useful research tool, which can potentially provide a rich and detailed, yet complex, account of data” (page 78). The six phases of thematic analysis were followed, based on Braun and Clarke [36,37]. In phase 1 (“Familiarizing yourself with the data”), the authors familiarized themselves with the interview questions and data, acknowledging any pre-existing knowledge and bias. This was accomplished by reading the responses multiple times, making notes, and discussing initial ideas. In phase 2 (“Generating initial codes”), initial codes were generated by both reviewers working together. The authors systematically worked through the interview questions, coding the content of the responses. This was accomplished with color coded sticky notes and a whiteboard. To ensure that evidence for each category could be revisited, the data were color coded for each participant. Examples of codes included ‘nutrition not a priority’, ‘status symbol’, and ‘value for money’. When a code appeared for the first time, it started a new category of data, and if the code appeared again, the category became more prominent as a theme. As this research sought to identify motivators and facilitators, the data for each of these questions were analyzed (and reported on) separately. In phase 3 (“Searching for themes”), the categories established through the interview coding were collated into potential themes by two authors. In phase 4 (“Reviewing themes”), the two authors checked the coding and themes to generate a thematic map. Throughout the process, any discrepancies were resolved by discussion. The thematic map was generated in Microsoft 365 Excel, Version 2502, where the codes, categories, and themes were clearly identified using a color-coding system. Phase 5 (“Defining and naming themes”) included ongoing analysis to ensure clear names and definitions existed for all themes. Phase 6 (“Producing the report”) included writing up the findings for this manuscript. The 15-point checklist, developed by Braun and Clarke [36], was used to ensure all steps were carried out. Descriptive responses that were direct (i.e., yes or no answers and rating scales), were quantitatively analyzed and did not contribute to the themes.

## 3. Results

### 3.1. Current Perceptions of Behaviors

**Figure 2:**
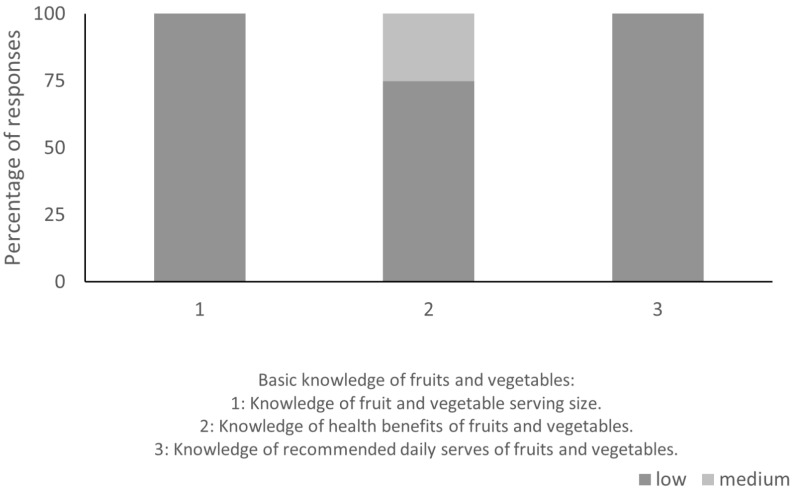
Percentage of participant responses for question 4.3 (knowledge related to fruits and vegetables). Components 1, 2, and 3 were answered using a rating scale (low, medium, and high).

Of the 18 interview questions, 5 reported data that were quantitatively analyzed. The results from the small group semi-structured interview were excluded from the quantitative analysis as not every participant responded. A total of 87.5% of participants thought that low fruit and vegetable consumption was an issue in Tonga. NCDs (85.7%), diabetes (25%), and obesity and weight (25%) were reported to be serious health concerns in Tonga, and 12.5% reported that overall health in their community was “good”. All participants agreed that diverse fruit and vegetable consumption is a priority for Tongan people and that communities are motivated to consume more fruits and vegetables. Perceptions of current knowledge related to fruits and vegetables were similar across serving size, health benefits, and recommended daily serves (question 4.3). Figure 2 outlines the percentages of the responses given for question 4.3; overall, knowledge was low across the three components.

### 3.2. Motivators

Several motivators of fruit and vegetable consumption were identified, including perceptions, preferences, and practices; consumption patterns; financial considerations; health and nutrition knowledge; availability and access; and production (further outlined in Table 2). The theme of perceptions, preferences, and practices included responses related to the use of luxury items, taste preferences, nutritional practices, adults’ and children’s preferences, and meat preferences. The theme of consumption patterns included responses related to traditional dietary patterns, fast food consumption, time poor and convenience factors, and special occasions. The theme of financial considerations included responses related to the income needed for a healthy lifestyle, price-hindering purchases, and prioritizing money-making over growing/cooking. The theme of health and nutrition knowledge included responses related to fruits and vegetables purchased for health reasons and how knowledge of disease increases fruit and vegetable consumption. The theme of access and availability included responses related to how gardens increase availability, fruits and vegetables only being eaten when available and accessible, increased access is needed for all communities, and access and availability for the outer islands. The theme of production included responses related to how community success encourages growing, the cost for production being too high, women interested in growing crops, and the overall interest level of growing crops consistently.

### 3.3. Facilitators

The facilitators of fruit and vegetable consumption were education; community engagement; environmental factors; sociocultural factors; food literacy skills; and accessibility (further outlined in Table 3). The theme of education included responses related to government awareness campaigns, knowledge and awareness, women facilitating learning, and education beginning with children. The theme of community engagement included responses related to group support increasing success, knowledge sharing leading to community engagement, and enjoying working together. The theme of environmental factors included responses related to land available for growing, different soil supporting or not supporting vegetable growth, and natural disasters/weather possibly destroying facilities. The theme of sociocultural factors included responses related to social trends, cultural norms, and encouragement through seeing other people change their behaviors. The theme of food literacy skills included responses related to the use of cooking facilities and appliances and knowledge and skills. The theme of accessibility included responses related to home and garden proximity increasing fruit and vegetable access and transportation. Interestingly, some of the themes were seen as both motivators and facilitators.

## 4. Discussion

This study aimed to explore the motivators and facilitators of fruit and vegetable intake in Tonga adults, thus contributing new and insightful data to the limited food choice behavior literature currently available for Tonga and the greater Pacific region. A variety of motivators and facilitators were identified, with some of these falling into both categories. Some motivating and facilitating determinants were also identified as factors that demotivate or impede fruit and vegetable intake for this participant group.

In Tonga, food holds cultural significance as it brings people together and can be used as a display of love [12,30]. For Pacific peoples, the traditional social values of food representing respect and politeness [12,30] strongly facilitates cultural identity and in turn their dietary patterns. In Tonga, fruits and vegetables can be costly and are therefore perceived as a symbol of status and may be reserved for special occasions [26]. The participants reinforced this as it was stated that social gatherings and special occasions may be the only time a household will consume fruits and/or vegetables due to financial and availability factors. Participants also indicated that there is cultural and social value in meat consumption, and it is considered very important in one’s diet. This is reflected in previous data which show that meat contributes 16% of the average dietary energy intake in Tongan diets compared to 5% from fruits and 2% from vegetables [38]. Similar but limited data are available for Polynesian countries, including Samoa [39] and Wallis and Futuna’s [40], but there are still significant gaps in the documented knowledge of the drivers of food choice and the consequent impact on diets in the region. This reinforces the critical need for more food and nutrition research.

Fruits and vegetables are part of a traditional Tongan diet, and it was reported by the participants that they enjoy eating these foods, even though it was acknowledged that fruit and vegetable intake is low. Taste and food preference were highlighted as a motivator of fruit and vegetable consumption for Tongan adults, and participants even reported this as an influence for children. The participants highlighted sweet, salty, and savory as traditional Tongan taste preferences, therefore having an influence on their decision-making when it comes to cooking and eating fruits and vegetables. It is common that the taste profiles of a westernized diet are often more desired, given the higher sugar and/or salt content, than the taste profiles of less processed and traditional foods, particularly for younger individuals [41]. The participants reflected this as they described how individuals (particularly youth) have been reported to prefer westernized food and consequently consume a low intake of fruits and vegetables. In this study, visual appearance and taste were reported to be key influences on younger individuals’ dietary intake; whilst for older people, cultural values and health benefits were reported to be driving factors for consumption. Research [1,42] reflects this finding, and studies in Asia [43] and New Zealand [44] have traced taste and food preference to generational factors as older generations tend to be anchored in their traditions, compared to younger people who are influenced by modern trends. Whilst the specific reasoning behind the difference in generational food choices was not investigated in this study, prior research justifies that younger individual’s food choice is more likely to be influenced by technology, brand marketing, influencer messaging, and social media usage [25,45]. Exploring, within the Tongan context, the relationship between taste, preference, and traditional values versus modern trends in further detail is warranted, as it may provide a more thorough understanding of the motivators of fruit and vegetable intake. Additionally, enhancing the understanding of the complexity of cultural values and social influences on food choice can contribute to the development of comprehensive nutrition and food policies and/or successful behavior changing nutrition and healthactivities.

Nutrition education and food literacy were identified as strong facilitators of fruit and vegetable intake. Food literacy can facilitate an increase in fruit and vegetable consumption through enhanced skills, knowledge, and behaviors related to planning, selecting, cooking, and eating [46]. Participants identified that nutrition education and food literacy skills are critical for understanding the relationship between nutrition and health and therefore developing nutritious food choice behavior patterns. The participants identified children as a key group for targeting health and nutrition educational awareness campaigns given children’s perceptions of food and that healthy behaviors are more likely to be established in childhood [4]. Women were also identified as a key target group by the participants as women are considered “responsible” for the nutrition and food intake of household members. The current literature emphasizes this as Pasifika mothers are identified as a strong influence on the household food environment as they facilitate the selection, preparation, and cooking of food [47]. To increase fruit and vegetable intake not only in women but subsequently their households, nutrition education should be supportive of women’s learning. It was identified that community engagement or working in a group is a successful method for implementing nutrition education amongst women but also for fostering an environment specifically for employing fruit and vegetable initiatives. The participants reported that Tongan people are “social beings” who enjoy working in a group environment (often where friendly rivalry promotes success and where participants “feel most engaged”) and discussed how working together can facilitate an environment where individuals are held accountable for improving their health. It was highlighted that Tongan women’s groups (i.e., handicraft or church groups) are interested in growing fruits and vegetables; however, they do not always have the opportunity to be involved in these activities. Group collaboration is an opportune forum for developing food literacy knowledge and skills; thus, nutrition education should be used as a facilitator for increasing fruit and vegetable intake in this demographic. To the authors knowledge, the theme of community engagement or working as a group has not been identified in the South Pacific-based literature as a facilitator for changing food choice behaviors.

The cost of purchasing fruits and vegetables was consistently mentioned throughout the interviews as both a motivator, facilitator, and barrier to consuming fruits and vegetables. According to the Tongan 2021 census, 38% of Tonga’s total population has a regular income [33] with the average monthly household income at 5145 Tongan Pa’anga (~3383 Australian dollars) [48]. Unaffordable fruit and vegetable prices are seen across the Pacific, as highlighted by the Household Expenditure Survey and by a study conducted in Vanuatu [49]. The Household Income and Expenditure Survey reported that Tongan people spent up to 27% of their household expenditure on fruits and vegetables [50]. Similarly, in Vanuatu it was found that lower socioeconomic households would have to spend up to 40% of their food budget on fruits and vegetables, which potentially reduced the intake of other essential food groups [49], highlighting a challenging dynamic between balancing a diverse diet (including micronutrient adequacy) and managing budgets. The increased cost of living has driven people to focus on money making (specifically handicrafts made by Tongan women) to generate an income [51]. This impact can be two-fold, as it can provide an increased income; however, as people are working more, they may be further inclined to purchase convenience foods or to spend large portions of their income on fruits and vegetables as they may not garden as often [51]. These financial challenges associated with healthy eating exacerbate the intake of less nutritious, cheaper foods, increasing the rate of DR-NCDs and poor food choices and behavior.

Whilst participants discussed that it is expensive to purchase fruits and vegetables, they also noted that it is costly to produce and grow crops. Subsistence farming has declined in recent decades; however, it is still a common method of sourcing food, generating income, and ensuring food security [52]. Food aid and home garden assistance are available in Tonga, and many communities appreciate the support of these programs. However, it was noted by participants that once the assistance is no longer available, it can be difficult to maintain these activities due to financial constraints. Across the Pacific Islands region, monetary factors associated with production and growing include insufficient access to crops and seedlings, limited land access, minimal and expensive farming equipment, and severe weather events [52]. It is well-known that the Pacific Islands region is exposed to more severe aspects of climate change (such as cyclones and tsunamis as experienced in Tonga in recent years). The unpredictability and severity of these events can cause unsustainable crop production and financial strain on communities, posing a threat to consistent and adequate fruit and vegetable intake [53] and overall diet quality.

This exploratory study was designed to provide insight into food choice behavior patterns; however, it provides scope for more diverse and larger food choice behavior studies in Tonga and the South Pacific more broadly. The findings in this study, whilst not representative of a large sample size and only representing those in Tongatapu, do provide an important initial insight into the complex dynamics between social, cultural, engagement, financial, and environmental determinants influencing the decision-making process of consuming fruits and vegetables in an adult Tongan population. The authors acknowledge that future or follow up work needs to include larger and more diverse samples, both geographically and socio-demographically, to further analyze the complexity of food choice behavior in Tonga.

### Limitations

A limitation of this study was the level of diversity of participants and the sample size; however, participant burden and data saturation were considered when making this decision. The authors acknowledge that the study findings may not represent all of Tonga. Both sexes were included to account for any differences in gendered experience and opinion. There was a risk of group thinking in the small group interview; however, measures were in place to limit this as much as possible. This was considered during analysis; thus, to limit any inconsistencies in the quantitative analysis, the small group responses were excluded for the following questions: 1.5, 2.1, 3.3, 3.5, and 4.3. A strength in conducting the interviews in Tongan communities at a small group or individual level, using a Tongan local and native speaker, is that the environment may have been more comfortable for the participants, prompting more honest responses than if the interviews were conducted by an English speaking, non-local person. It is also noted that this study was conducted at one point in time, and most of the participants were from Tongatapu (and one participant was from ‘Eua); thus, seasonality, economic fluctuation, and location may need to be considered when looking at the results. This does, however, provide scope for further research to determine if these motivators and facilitators are relevant during all seasons, economic situations, and across all inhabited Tongan islands.

## 5. Conclusions

This exploratory study investigated the motivators and facilitators of fruit and vegetable intake in Tonga adults. Semi-structured interviews were conducted in Tongan and were analyzed using reflexive thematic analysis. A total of 12 themes were identified and categorized into motivating and facilitating determinants. The results from this study are intended to inform future dietary intake studies in Tonga as well as provide context for further research in this area of food choice and behavior. Whilst they are specific to Tongan communities, some of the results are reflective of the previous literature in other Pacific Island countries and provide new information to fill in gaps that are missing for Tonga specifically. This information can inform governments, policymakers, and local NGOs with up-to-date contextually relevant information for improving and supporting health and nutrition in Tonga. This work was important as it identified research areas to be explored further to develop a comprehensive understanding of the complexity behind food choice behaviors in the Pacific region.

## Figures and Tables

**Table 1:** Semi-structured interview guide.

1	Demographic/Validation of the issue
1.1	Are you over the age of 18? If no, thank them for their time and do not proceed with interview.
1.2	Do you consent to participating in the interview? If no, thank them for their time and do not proceed with the interview.
1.3	If applicable: community name
1.4	What is your status/position within the community? What is your role?
1.5	Do you think low fruit and vegetable consumption is an issue in your community? Why or why not?
2	Audience’s current behavior (examples of prompting questions)
2.1	What health problems are [you/Tongan communities] currently concerned about?
2.2	What specific behaviors are [you/Tongan communities] engaging in that contribute to low * consumption of fruits and vegetables?
2.3	What are some practices that [you/Tongan communities] are engaging in that positively contribute to increasing fruit and vegetable consumption? What can be done to strengthen these practices to increase fruit and vegetable intake?
3	Motivators (examples of prompting question)
3.1	What are the motivators for eating fruit and vegetables for [yourself/Tongan communities]?
3.2	What attitudes do [you/Tongan communities] have towards eating fruits and vegetables?
3.3	Yes or no, is it a priority for [yourself/Tongan communities] to consume a diverse range of fruits and vegetables? Why or why not?
3.4	What social barriers stop [yourself/Tongan communities] from achieving an increase in fruit and vegetable consumption?
3.5	Are [you/Tongan communities] motivated to consume more fruits and vegetables?
4	Facilitators (examples of prompting question)
4.1	a) What facilitates consuming fruits and vegetables on an individual and community level? b) What skills do the members of your community have that facilitate consuming fruits and vegetables?
4.2	Does the physical environment support an increase in fruit and vegetable consumption?
4.3	What level of nutrition knowledge does your community have about: (self-perceived as low, medium, or high) c) Serving sizes of fruits and vegetables? d) Health benefits of eating a diverse range of fruits and vegetables e) The recommended serves for fruits and vegetables per day?
4.4	Would your community members be willing to engage in practices/workshops that focus on increasing food literacy? What members of the community do you think would be most interested?
4.5	How would social norms and patterns influence the success of activities that are designed to increase the consumption of fruits and vegetables?

* Low based on not meeting food based dietary guidelines.

**Table 2:** Motivating themes of fruit and vegetable consumption including a description and indicative quote from the interviews.

Motivator	Description	Indicative Quote
* Key theme: Perceptions, preferences, practices.		
Luxury items.	Fruits and vegetables can be perceived as a luxury item or a status symbol; mostly consumed during special occasions.	“Fruits and vegetables are also seen as a status symbol and reserved for special occasions.”
Taste preference.	Taste preferences include sweet, salty, and savory.	“Our tastes always lean to either salty and savory or sweet so it’s hard to understand that an all-vegetable dish would taste good.”
Nutrition not practiced.	Although the health benefits of nutrition are understood, healthy eating is not actively practiced.	“It’s just harder to do it in practice for many reasons but mostly because it’s not something that takes up a lot of our thinking.”
Adults’ vs. children’s preferences.	Adults may include fruits and vegetables into their diets for health reasons, compared to children who may or may not eat fruits and vegetables for taste and visual appearance.	“Vegetables are more attractive for the adults because they know more about nutritional value verses the kids who would like things for its taste and appearance.”
Preference for meat.	Meat is enjoyed and consumed daily in most meals. There is cultural and social value in meat consumption, and it is considered very important for family intake.	“It’s very clear that they won’t give up meat to get veggies.”
Key theme: Consumption patterns		
Traditional diets.	There is significance in consuming traditional diets/foods in Tongan culture. Traditional diets do not include a vast variety of fruits and vegetables.	“… many households simply appreciate eating fruits that have always been a part of our diets…”
Fast food consumption.	Fast food has become increasingly popular for its taste and convenience.	“Fast food joints like fried chicken and BBQ stands are popular because it’s fast and convenient.”
Time poor and convenience factors.	Time availability and the role of convenience foods have changed dietary patterns. This is seen in working people who may be time poor and therefore are consuming convenience foods more often.	“The eating patterns have changed… working family members have shifted their diets to eating more imported, convenient on the go foods like meat pies.”
Special occasions.	Vegetables are included in meals during special occasions to bulk up the meals to feed more people, for cultural reasons and for financial reasons.	“They will buy [fruits and vegetables] for church functions and that stuff but for normal consumption of the family—no.” “… very expensive and most times we only buy it for special occasions.”
* Key theme: Financial considerations		
Income needed for healthy lifestyle.	To purchase healthy foods and engage in healthy practices (i.e., gardening) requires a stable and sufficient income. Some communities do not have a stable income and/or have to prioritize putting money towards other aspects of their lives.	“It’s been very difficult to continue farming simply because it’s very expensive… not many of us here in the community have regular incomes or have jobs.”
Price hinders purchase.	Healthy eating can be unachievable due to the price. Some perceive the product for the price it is sold at as not being ‘value for money’.	“Financial constraints can hinder many families from eating a lot of fruits and vegetables, either it is too expensive, or they do not have the means to buy fruits and vegetables.”
Prioritizing money making over growing/cooking.	Women prioritize handicraft work to generate income for their household over gardening and/or cooking in the kitchen. Generating income allows the women to purchase fruits and vegetables	“Women prioritize working to produce handicrafts for livelihoods… selling handicrafts makes more money… enough money to buy fruits and vegetables.”
Key theme: Health and nutrition knowledge		
Fruits and vegetables purchased for health reasons.	The benefits of healthy eating are understood and encourage fruit and vegetable purchasing and consumption.	“People eat fruits and vegetables because they know that it’s good for their health. That’s what pushes them to continue eating it and buying.”
Knowledge of disease increases fruit and vegetable consumption.	Preventable DR-NCDs and associated death is a major motivator to increasing fruit and vegetable intake. Understanding the nutritional value and its benefits being linked to illness and disease also encourages intake of healthy foods.	“Sometimes it’s often the death of someone they know or is near to and when they find out about cause of death being preventable then there is a huge shift to change.”
* Key theme: Access and availability		
Gardens increase availability.	Having either home or community gardens increases the availability of fruits and vegetables for many communities.	“If we have access to [fruits and vegetables] like I do with this vegetable garden, it makes everything that much easier.”
Fruits and vegetables only eaten when available and accessible.	People enjoy eating fruits and vegetables; however, they can only be eaten when they are available and accessible.	“There is always a need to have it every day, unfortunately it is only what we can have that is available to us at the time.”
Increased access is needed for all communities.	Communities are provided aid in accessing fruits and vegetables. This is only for some communities, and it has been identified that all communities should receive assistance.	“We need to make vegetables and fruits more accessible to all communities.”
Outer islands access and availability.	Access can be difficult for outer island communities. Assistance is given for growing; however, most purchasing of fruits and vegetables is conducted on the mainland.	“I see that when people from outer islands travel to mainland and see fresh fruits and vegetables they buy as much as they can.”
Key theme: Production		
Community success encourages growing.	Communities can often be motivated by other’s success. Friendly competition also encourages change and fruit and vegetable production.	“For community groups, they often times are motivated by looking at the success of other community groups—sort of a friendly rivalry that starts but is beneficial for many.”
Cost for production is too high for local produce (equipment).	Producing certain foods and crops can be expensive in addition to costly industrial equipment.	“The problem is the cost of production is too high to produce locally.”
Women are interested in growing crops.	Women are interested in gardening and participating in growing programs. It has been acknowledged that women are grateful for programs and assistance as they may not have the same opportunities to grow crops on their own.	“From my work experience there is a huge interest by women groups to grow fruit trees and vegetable gardens.”
Level of interest in growing crops consistently.	When assistance is provided to communities, the interest in growing fruits and vegetables exists; however, this can dimmish once the assistance ends and the work becomes too much for communities.	“Others find it too much work and become disinterested.”

* This was found in facilitators as well.

**Table 3:** Facilitating themes of fruit and vegetable consumption including a description and indicative quote from the interviews.

Facilitator	Description	Indicative Quote
Key theme: Education		
Government awareness campaigns.	The Ministry of Health has many programs that are delivered through radio, schools, and TV that address nutrition and health.	“Awareness campaigns that national government runs through the media make people aware of good nutrition.”
Knowledge and awareness.	Nutrition and health knowledge can encourage healthy eating and food choice both at an individual and community level.	“What can help people eat more fruits and vegetables is to have more knowledge, understanding, and awareness of nutritional value.”
Women facilitating learning.	Women groups can facilitate learning from one another. Women are interested in developing their skills and sharing knowledge.	“Women groups like to have programs that facilitate group meetings and learning from each other.”
Education begins with children.	Education begins with children. Healthy dietary patterns established in childhood are likely to carry into adulthood.	“I think it’s important that awareness programs run all the time and that we start from the young kids at school and teach them about nutrition.”
Key theme: Community engagement/working in groups		
Group support increases success.	Group work can encourage collaboration of knowledge and can hold individuals accountable for staying on track with dietary goals.	“Working in groups is something that can build on the success of the activities… being in a group can drive for more action and ensure people continue the work.”
Sharing knowledge engages community.	Working together provides a forum for sharing knowledge, collaboration of ideas, and expanding skill sets.	“… knowledge about diverse range of fruits and vegetables are shared constantly… this is a skill in which can help community members to try new things, gain more knowledge…”
Tongans enjoy working together.	Group work can create a positive environment for learning, growing, and consuming fruits and vegetables.	“Working individually sometimes doesn’t help because we enjoy working together.”
Key theme: Environmental factors		
Land available for growing.	There is tax allotment land for growing fruits and vegetables in Tonga.	“There is arable land available for many communities to grow fruits and vegetables.”
Different soil may or may not support vegetable growth.	Soil quality may or may not be supportive of vegetable growth across the various islands.	“This property is the tax allotment so the soil here is perfect for growing.” “Vava’u is different—the soil is mostly rocky and is not great for planting vegetables.”
Natural disaster/weather can destroy facilities.	Tonga is prone to cyclones and tsunamis, and most coastal communities are low-lying. Some infrastructure and cooking facilities have been adapted for these events; however, most outdoor kitchens are not built to withstand natural disasters or strong weather.	“These small outdoor kitchens aren’t built to withstand strong winds so they would usually get demolished in strong winds.”
Key theme: Sociocultural factors		
Social trends.	Tongans are influenced by key community members (mothers in households, community leaders, church leaders, etc.) and social trends.	“Influence of key community members can help support and strengthen uptake of nutrition education/awareness to community members.”
Cultural norms.	Tongan people value traditions and customs, and cultural values can encourage growing fruits and vegetables. Religious teachings can also advocate for prioritizing health.	“If there are more people gardening and growing more at home, then through our cultural values of sharing with each other will lead to supporting the efforts of having people eat more fruits and vegetables.”
Encouragement through seeing change.	Change can come from watching others change and succeed.	“… people telling them these success stories—it motivates them to try those new things.” “People change their attitudes and behavior when they see change in others.”
* Key theme: Food literacy skills		
Cooking facilities and appliances.	All households in Tonga have indoor kitchen facilities, some have additional outdoor kitchens. Outdoor kitchens utilize ground ovens and wood fires, whereas indoor kitchens utilize propane gas ovens. Electronic appliances are also used; however, there is a desire for improved cooking equipment.	“In regard to households having the right facilities that can help in food preparation, well just about every household has a kitchen.” “I wish I could improve a lot of cooking tools.”
Knowledge and skills.	Knowledge and skills can include nutritional compositions, serving sizes, food safety, reading recipes, identifying cooking utensils, cooking, and storing ability.	“I think what is really important is that the people have the knowledge around nutritional value of foods, … have the skills to know how to identify certain vegetables and fruits, be able to grow them organically and how to prepare it for the whole family.”
* Key theme: Accessibility		
Home and garden proximity increases fruit and vegetable access.	The close proximity of the house and gardens allows for regular access to fruits and vegetables.	“The distance between the homes and the vegetable gardens for women are relatively close so it’s easier for them to access more fruits and vegetables on a regular basis.”
Transportation.	Vehicles are needed for accessing fruits and vegetables from markets in Tongatapu. Rural/outer islands communities use boats to travel to the main island for fruits and vegetables.	“For most people they have vehicles, the outer islands have boats, it’s all about access.”

* This was found in motivators as well.

## Data Availability

Please contact the corresponding author to request data.

## Abbreviations

The following abbreviations are used in this manuscript:

DR-NCDs	Diet-related non-communicable diseases
NGO	Non-government organization
